# Case report: A patient with Delayed Sleep-Wake Phase Disorder and Optic Nerve Hypoplasia treated with tasimelteon: a case study

**DOI:** 10.3389/fnins.2023.1287514

**Published:** 2023-11-14

**Authors:** Sandra P. Smieszek, Alyssa R. Kaden, Caroline E. Johnson, Jennifer L. Brzezynski, Changfu Xiao, Christos M. Polymeropoulos, Gunther Birznieks, Helene A. Emsellem, Mihael H. Polymeropoulos

**Affiliations:** ^1^Vanda Pharmaceuticals Inc., Washington, DC, United States; ^2^The Center for Sleep & Wake Disorders, Chevy Chase, MD, United States; ^3^Department of Neurology, George Washington University, Washington, DC, United States

**Keywords:** biological rhythms, circadian rhythm disorders, clinical trials research, sleep/wake mechanisms, Delayed Sleep-Wake Phase Disorder, delayed sleep, night owl, Optic Nerve Hypoplasia

## Abstract

We present a case of an adult female diagnosed with Delayed Sleep-Wake Phase Disorder (DSWPD) and Optic Nerve Hypoplasia (ONH), with a confirmed delayed Dim Light Melatonin Onset (DLMO), who reports the inability to fall asleep at their desired bedtime and obtain adequate sleep nightly, despite the ability to have a full night’s sleep when not required to be up at a specific time for societal requirements. The participant was enrolled in an 11-month Open-Label Extension (OLE) following the randomized portion of a clinical study and was successfully treated with tasimelteon. DSWPD symptoms were resolved, and their previously delayed sleep-wake cycle was advanced.

**Clinical trial registration:**

https://clinicaltrials.gov/ct2/show/NCT04652882, identifier NCT04652882.

## Introduction

Delayed Sleep-Wake Phase Disorder (DSWPD) is a Circadian Rhythm Sleep-Wake Disorder (CRSWD) affecting the timing of sleep, peak period of alertness, core body temperature rhythm, and other circadian rhythms, when compared to the general population and relative to societal requirements (Littner et al., 2005). DSWPD is characterized by sleep initiation insomnia when attempting sleep at conventional times and difficulty waking at the required time for daytime commitments (American Academy of Sleep Medicine, 2014). Patients with DSWPD experience difficulty shifting their sleep episode to an earlier time, often resulting in social and occupational dysfunction. When a shift to an earlier time is not needed (work/school-free days), DSWPD patients may sleep for an extended period of time, often more than 8 h, albeit occurring at later times compared to societal conventions. There is no approved treatment for DSWPD, and the American Academy of Sleep Medicine (AASM) recommends morning light exposure (Morgenthaler et al., 2007). This treatment is well tolerated, but more potent and easier to adhere to treatments are needed.

Tasimelteon is a melatonin receptor agonist that demonstrates high affinity for both the human melatonin MT_1_ and MT_2_ receptors. In binding to the MT_1_ and MT_2_ receptors, tasimelteon acts by entraining circadian sleep phase timing and has been shown to improve nighttime sleep as well as daytime sleepiness and functioning. Tasimelteon is the first and only approved medicine to treat a CRSWD and is being assessed for a new circadian rhythm indication in DSWPD.

A multicenter, double-blind, randomized clinical study to evaluate the effects of tasimelteon versus placebo in DSWPD participants is ongoing. This study consists of screening and treatment phases, followed by an 11-month Open-Label Extension (OLE) phase. We report our first completed participant from OLE phase, who was successfully treated with tasimelteon over approximately 11 months.

During the OLE phase, participants answer daily sleep diaries and are instructed to take one dose of tasimelteon, 60 min prior to their desired bedtime. Desired bedtime is determined by the participant, and is defined as the time the participant would need to go to bed the night before a commitment, in order to feel fully rested in the morning. The objective of the OLE phase is to explore the long-term safety and efficacy of daily dosing with tasimelteon over 11 months of treatment. As this DSWPD study is currently ongoing, conclusions cannot be made about treatment assignment during the double-blind treatment phase. The study was approved by human research Institutional Review Boards (IRBs) at all participating institutions. All participants provided written informed consent and were provided a copy of the signed consent form before any screening procedures occurred.

## Report of case

We present a case of a 24-year-old female diagnosed with DSWPD and Optic Nerve Hypoplasia (ONH), with a confirmed delayed Dim Light Melatonin Onset (DLMO), who reports the inability to fall asleep at their desired bedtime and the ability to have a full night’s sleep when not required to be up at a specific time for societal requirements.

Prior to enrollment in the study, the participant reported delayed sleep since childhood and recalled feeling better when they slept in later. During high school, their sleep schedule on weekdays consisted of going to bed at midnight and waking up at 06:00 in the morning for school in order to arrive by 07:00. Due to their late sleep onset and early wake time, they reported being always exhausted and found the weekends particularly helpful, as they were able to go to bed between midnight and 01:00 and sleep in. In college, when they had more flexibility with their schedule, they stayed up later and avoided classes before noon, following a typical sleep schedule of 03:00 in the morning to noon or later. When the participant entered the workforce after college, their schedule became fixed again, with their job starting and ending at 09:00 and 17:00, respectively. They stated that they generally remained phase delayed after college, having restricted sleep during the work week due to their schedule, and unrestricted sleep on the weekends due to being able to sleep in late.

The participant also reported typically taking sleep medications five times per week, before participation in the study. They took Natrol (melatonin) 5 mg, fast-dissolving tablets orally from August 2021 to October 2021, on Sunday through Thursday nights before work the following day. They did not take melatonin at a single fixed time, but reported taking it approximately 30–60 min prior to their habitual bedtime, resulting in a sleep onset between 00:30–02:00. Overall, the participant stated that the melatonin was helpful, but reported nocturnal awakenings throughout the night when they took the drug. They recall that taking a second dose of melatonin helped them fall back to sleep when they experienced these nocturnal awakenings. They were able to wake up at 07:30–09:00 in the morning for work by using multiple alarms; however, they were tired for the rest of the day. The participant also reported feeling more fatigued during the day when they took a second dose of melatonin, versus taking a single dose at bedtime.

The participant enrolled in the DSWPD study and was diagnosed with DSWPD according to the International Classification of Sleep Disorders, Third Edition (ICSD-3) (American Academy of Sleep Medicine, 2014). During clinical interviews, the participant reported that they got into bed with the intention of going to sleep at 23:30 and reported a typical sleep latency of 90 min to 2 h. The participant reported waking up at 08:00 on weekdays and always feeling tired despite their amount of sleep. On weekends, they reported going to bed between 01:00 and 02:00 and falling asleep immediately. They would wake up naturally around noon and feel completely rested. The participant’s self-reported habitual sleep onset was 01:00, habitual bedtime 23:30, and desired bedtime 23:00, per the DSPD Screening Questionnaire (Smith et al., 1989).

The participant did not report using tobacco or frequently consuming alcoholic beverages. They are employed 40 h/week with all work hours falling between 07:00 and 22:00 (typical start time of 09:00 and end time of 17:00). They reported they are “definitely an evening type,” with a total score of 24 on the Morningness-Eveningness Questionnaire (MEQ). The participant’s total sleep disturbance score was 62 and their sleep-related impairment score was 59, according to the Patient-Reported Outcomes Measurement Information System (PROMIS) questionnaires. The participant had a 12-lead electrocardiogram (ECG) performed, which was deemed “Abnormal Not Clinically Significant” by the Investigator and a Body Mass Index (BMI) of 20.9. They did not report a history of psychiatric disorders in their medical history, when asked by site staff.

During the screening phase of the study, the participant’s DLMO was assessed and determined to be delayed at 23:14, which is significantly later than that of population controls (Supplementary Figure 1). Delayed DLMO is defined as DLMO occurring after or within 30 min before desired bedtime, and after 21:30 (Murray et al., 2017). DLMO was calculated and defined as the clock time when the melatonin concentration exceeded the mean of three low consecutive values, plus twice the standard deviation of these points. The DLMO assessment was conducted at home, and consisted of eight scheduled saliva collections to be performed beginning from 5 h before bedtime until 3 h after bedtime. The DLMO assessment bedtime during screening was the participant’s habitual bedtime. Participants were instructed to sit in dim light conditions during the DLMO sampling period and remain seated with limited movement for 20 min before each sample. Additionally, the participant was instructed to wear blue-light blocking glasses or sunglasses during the DLMO assessment period. No food or beverages other than water were to be consumed within 10 min of each sample. This assessment was distributed at screening and collected at the next visit.

The participant’s medical history includes astigmatism and nearsightedness in both eyes (prescription of −3.50 in the right eye and −4.00 in the left eye), which they were prescribed glasses and contacts for. This led to their bilateral Laser-Assisted *In Situ* Keratomileusis (LASIK) eye surgery in September 2019. The participant reported no abnormal visual history during their childhood. In December 2018, the participant had an optical coherence tomography (OCT) scan done with abnormal results, which prompted them to be seen by an ophthalmologist. They were then diagnosed with bilateral ONH, a medical condition arising from the underdevelopment of the optic nerve. The participant reported no difficulties with their visual acuity, or light or color perception abnormalities. There is no significant or known family sleep or visual history. Neither of the participant’s parents are “night owls”; however, the participant’s adult sibling is severely phase delayed and sleeps from the early morning hours until 12:00–13:00.

**FIGURE 1:**
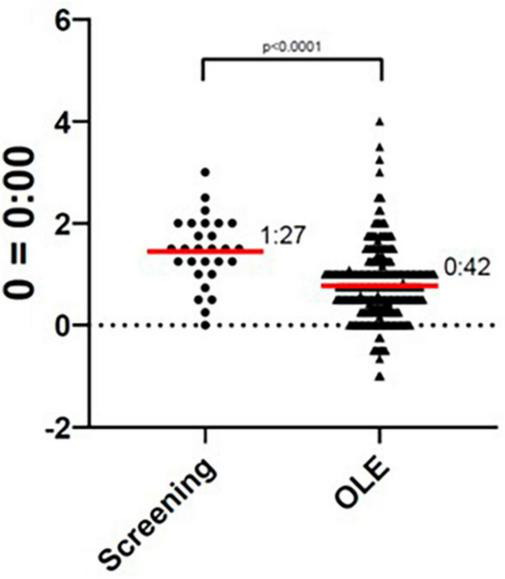
Sleep onset during screening and Open-Label Extension (OLE). The sleep onset time was significantly improved during the OLE phase as compared to the screening phase (*p* < 0.0001).

**FIGURE 2:**
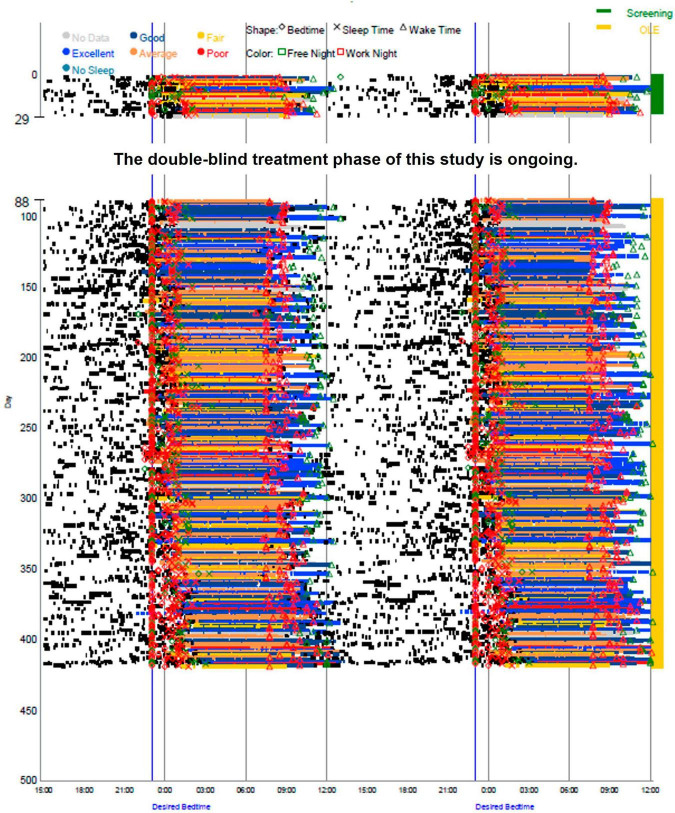
Sleep diary data during screening and Open-Label Extension (OLE). Sleep timing (sleep onset, duration, and wake time) was measured by an electronic daily sleep diary over the entire study, screening (green) and OLE (yellow).

The participant’s average sleep onset was 01:27 during screening and 00:42 during the OLE phase, which was a significant improvement (*p* < 0.0001), as seen in Figure 1. At screening, the participant reported their symptoms as moderate (3) on the Patient Global Impression of Severity (PGI-S). On average, during the OLE, the participant reported their symptoms as mild (2) on the PGI-S and much improved (2) on the Patient Global Impression of Change (PGI-C). Additionally, the Investigator reported their symptoms as much improved (2) on the Clinical Global Impression of Change (CGI-C), on average during the OLE. The participant’s sleep diary data during screening and OLE are shown in Figure 2. Daily sleep quality was assessed in the diary, as “Excellent,” “Good,” “Average,” “Fair,” or “Poor.” The reported sleep quality significantly improved during OLE, as compared to screening.

During their time in the study, there were no significant changes in their physical examinations, vital signs, ECGs, or BMI, with no suicidal ideation or behaviors. Clinical laboratory parameters (hematology, chemistry, and urinalysis) were consistent throughout the study.

## Discussion

The participant reported tasimelteon to be extremely helpful and that they were able to “live a normal life” during the OLE phase. The participant was able to go to bed at midnight and wake up at 07:30–09:00 for work. The participant’s daytime sleepiness was significantly improved, and they reported no nocturnal awakenings while taking tasimelteon, as opposed to melatonin. The participant stated that on weekends, they could “feel” the tasimelteon, and were able to maintain a steady bedtime throughout the OLE, despite sleeping in late on some weekend mornings. During the OLE phase, the participant’s DSWPD symptoms were resolved, and their previously delayed sleep-wake cycle was advanced.

After their participation in the OLE phase was completed, they stated that their entrainment to the 00:00–00:30 bedtime progressively diminished, and they now go to bed at 01:00–02:00. The participant’s daytime sleepiness has also regressed back to its state prior to the OLE phase. The participant stated that they would like to continue taking tasimelteon, as their daytime energy and alertness was best while on this study drug.

The participant’s comorbidities of DSWPD and bilateral ONH are of interest, due to their possible association. ONH is the most common congenital optic nerve anomaly and could indicate lack of proper optic response to light. Abnormal rest-activity rhythmicity patterns are present in 30% of children with ONH (Rivkees et al., 2010). *math5* is a critical regulator of retinal ganglion cell development. *math5*^–/–^ mice show severe ONH and no entrainment to light/dark cycles, whereas heterozygous mice show normal entrainment to both 12-hour light/dark cycles and a 1-hour skeletal photoperiod (Wee et al., 2002). While retinal ganglion cell input is not necessary for the development of a free-running circadian timekeeping system in the suprachiasmatic nucleus, it is important for both photic entrainment and determination of the free-running period.

Further research is necessary to characterize the relationship between light responsivity and phenotype impact such as ONH on circadian rhythms and sleep. Consequential variants in melanopsin (*OPN4*), a blue light-sensitive opsin-type G-protein coupled receptor, were previously associated with an increased risk of developing seasonal affective disorder (Chellappa, 2021). Melanopsin-dependent phototransduction was also reported to be impaired in DSWPD and sighted Non-24-Hour Sleep-Wake Disorder (Abbott et al., 2021). This participant is not a carrier of the variable number of tandem repeat (VNTR) *PER3*^4/4^ genotype, associated with delayed sleep patterns, nor the *CRY1* splicing variant (Patke et al., 2017). This patient has been determined to carry two 5′ UTR region variants in the Atonal BHLH Transcription Factor 7 (ATOH7) gene known to be associated with ONH, rs61854782 and rs7916697. ATOH7 is expressed in retinal progenitor cells and has a crucial role in retinal ganglion cell development. No other predicted loss-of-function mutations within circadian genes were identified.

This case illustrates the general positive effect of tasimelteon on a participant diagnosed with DSWPD, based on earlier sleep onset shift during an open-label dosing period compared to screening, overall improvement of PGI-S responses during OLE compared to screening, and PGI-C responses over time. It also provides the opportunity for research into ONH and its relationship with DSWPD, and potentially sighted Non-24-Hour Sleep-Wake Disorder.

## Data availability statement

The original contributions presented in this study are included in the article/Supplementary material, further inquiries can be directed to the corresponding author.

## Ethics statement

The studies involving humans were approved by the Advarra Institutional Review Board. The studies were conducted in accordance with the local legislation and institutional requirements. The participants provided their written informed consent to participate in this study.

## Author contributions

SS: Conceptualization, Formal analysis, Investigation, Writing – original draft. AK: Investigation, Writing – original draft. CJ: Investigation, Writing – original draft. JB: Investigation, Writing – original draft, Writing – review and editing. CX: Data curation, Formal analysis, Writing – review and editing. CP: Conceptualization, Investigation, Writing – review and editing. GB: Conceptualization, Investigation, Writing – original draft. HE: Investigation, Writing – review and editing. MP: Conceptualization, Funding acquisition, Investigation, Writing – review and editing.

## Supplementary material

Supplemental Figure 1 Screening salivary Dim Light Melatonin Onset (DLMO). The screening DLMO of this participant is significantly later than that of population controls, occurring at 23:14.
