# Corrigendum: Case report: A patient with Delayed Sleep-Wake Phase Disorder and Optic Nerve Hypoplasia treated with tasimelteon: a case study

**DOI:** 10.3389/fnins.2023.1344915

**Published:** 2024-01-08

**Authors:** Sandra P. Smieszek, Alyssa R. Kaden, Caroline E. Johnson, Jennifer L. Brzezynski, Changfu Xiao, Christos M. Polymeropoulos, Gunther Birznieks, Helene A. Emsellem, Mihael H. Polymeropoulos

**Affiliations:** ^1^Vanda Pharmaceuticals Inc., Washington, DC, United States; ^2^The Center for Sleep & Wake Disorders, Chevy Chase, MD, United States; ^3^Department of Neurology, George Washington University, Washington, DC, United States

**Keywords:** biological rhythms, circadian rhythm disorders, clinical trials research, sleep/wake mechanisms, Delayed Sleep-Wake Phase Disorder, delayed sleep, night owl, Optic Nerve Hypoplasia

In the published article, there was an error. (There was an omission).

A correction has been made to **Discussion**, Paragraph 4. This sentence previously stated:

“This participant is not a carrier of the variable number of tandem repeat (VNTR) *PER*3^4/4^ genotype, associated with delayed sleep patterns, nor the *CRY1* splicing variant (Patke et al., 2017). No other predicted loss-of-function mutations within circadian genes were identified.”

The corrected sentence appears below:

“This participant is not a carrier of the variable number of tandem repeat (VNTR) *PER*3^4/4^ genotype, associated with delayed sleep patterns, nor the *CRY1* splicing variant (Patke et al., 2017). This patient has been determined to carry two 5′ UTR region variants in the Atonal BHLH Transcription Factor 7 (ATOH7) gene known to be associated with ONH, rs61854782 and rs7916697. ATOH7 is expressed in retinal progenitor cells and has a crucial role in retinal ganglion cell development. No other predicted loss-of-function mutations within circadian genes were identified.”

The authors apologize for this error and state that this does not change the scientific conclusions of the article in any way. The original article has been updated.
