# Supplementary figures and images for: Case report: A patient with Delayed Sleep-Wake Phase Disorder and Optic Nerve Hypoplasia treated with tasimelteon: a case study
Source: Front Neurosci. 2023 Nov 14;17:1287514. doi: 10.3389/fnins.2023.1287514 (PMC10682171; doi:10.3389/fnins.2023.1287514)

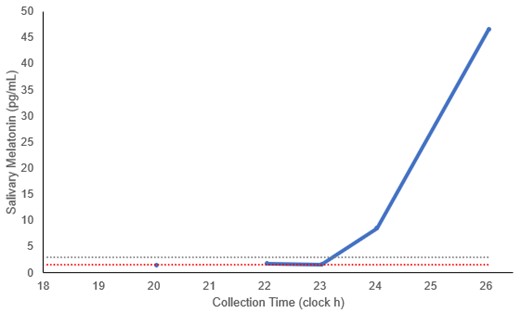

Supplement: Supplemental Figure 1 — Screening salivary Dim Light Melatonin Onset (DLMO). The screening DLMO of this participant is significantly later than that of population controls, occurring at 23:14. [file Image_1.JPEG]
