# Supplementary material for: Case report: A patient with Delayed Sleep-Wake Phase Disorder and Optic Nerve Hypoplasia treated with tasimelteon: a case study
Source: Front Neurosci. 2023 Nov 14;17:1287514. doi: 10.3389/fnins.2023.1287514 (PMC10682171; doi:10.3389/fnins.2023.1287514)
Supplement: Supplementary file 2 [file Data_Sheet_1.docx]

**Clinical Trial Registration**

**Trial Name**

A multicenter, double-blind, randomized study to evaluate the effects of tasimelteon vs. placebo in participants with Delayed Sleep-Wake Phase Disorder (DSWPD)

**ClinicalTrials.gov Identifier**

NCT04652882

**URL**

<https://clinicaltrials.gov/ct2/show/NCT04652882>

**Statement of Significance**

Tasimelteon is being evaluated in an ongoing clinical study to treat participants diagnosed with Delayed Sleep-Wake Phase Disorder (DSWPD). As there is currently no approved treatment for DSWPD, diagnosed patients have limited options. This study not only highlights a potential treatment option for patients with DSWPD, but lends credence to further research on its relation to Optic Nerve Hypoplasia (ONH) and potential circadian aberrations such ocular phenotypes may lead to.
